# Experiences in implementation and publication of operations research interventions: gaps and a way forward

**DOI:** 10.7448/IAS.19.5.20842

**Published:** 2016-07-20

**Authors:** Samuel Kalibala, Godfrey B Woelk, Stephen Gloyd, Nrupa Jani, Lynnette Kay, Avina Sarna, Jerry Okal, Charity Ndwiga, Nicole Haberland, Irit Sinai

**Affiliations:** 1 HIVCore/Population Council, Washington, DC, USA; 2 Elizabeth Glaser Pediatric AIDS Foundation, Washington, DC, USA; 3 Department of Global Health, University of Washington, Seattle, WA, USA; 4 Retrak Ethiopia, Addis Ababa, Ethiopia; 5 Palladium, Washington DC, USA

**Keywords:** operations research, implementation research, intervention fidelity, process evaluation, intervention publication

## Abstract

**Introduction:**

According to UNAIDS, the world currently has an adequate collection of proven HIV prevention, treatment and diagnostic tools, which, if scaled up, can lay the foundation for ending the AIDS epidemic. HIV operations research (OR) tests and promotes the use of interventions that can increase the demand for and supply of these tools. However, current publications of OR mainly focus on outcomes, leaving gaps in reporting of intervention characteristics, which are essential to address for the utilization of OR findings. This has prompted WHO and other international public health agencies to issue reporting requirements for OR studies. The objective of this commentary is to review experiences in HIV OR intervention design, implementation, process data collection and publication in order to identify gaps, contribute to the body of knowledge and propose a way forward to improve the focus on “implementation” in implementation research.

**Discussion:**

Interventions in OR, like ordinary service delivery programmes, are subject to the programme cycle, which continually uses insights from implementation and the local context to modify service delivery modalities. Given that some of these modifications in the intervention may influence study outcomes, the documentation of process data becomes vital in OR. However, a key challenge is that study resources tend to be skewed towards documentation and the reporting of study outcomes to the detriment of process data, even though process data is vital for understanding factors influencing the outcomes.

**Conclusions:**

Interventions in OR should be viewed using the lens of programme evaluation, which includes formative assessment (to determine concept and design), followed by process evaluation (to monitor inputs and outputs) and effectiveness evaluation (to assess outcomes and effectiveness). Study resources should be equitably used between process evaluation and outcome measurement to facilitate inclusion of data about fidelity and dose in publications in order to enable explanation of the relationship between dosing and study outcomes for purposes of scaling up and further refinement through research.

## Introduction

HIV operations research (OR) has been defined as a process of identifying and solving programme problems with the goal of increasing the efficiency, effectiveness, quality, availability, accessibility and acceptability of services [1]. In 2008, The Global Fund to Fight AIDS, Tuberculosis and Malaria, United States Agency for International Development (USAID), World Health Organization (WHO), Joint United Nations Programme on HIV/AIDS (UNAIDS) and the World Bank published a framework for HIV operations and implementation research [2]. That publication states that OR represents implementation research and is defined as any research producing practically usable knowledge that can improve programme implementation regardless of the type of research design, methodology or approach.

Building on this definition, PEPFAR's implementation science (IS) framework describes IS as the study of methods to improve the uptake, implementation and translation of research findings into routine and common practices [3]. Thus, although terminology referring to implementation or OR may vary depending on the context, the main intent is to examine health systems management and sociocultural, economic and behavioural factors that either exist as bottlenecks or that could be tested to improve service delivery and uptake [2].

According to UNAIDS, the world currently has a good collection of proven HIV prevention, treatment and diagnostic tools, which, if scaled up, can lay the foundation for ending the AIDS epidemic [4]. The effectiveness of these tools is often dependent on a number of operational issues on the demand side (e.g., the health care-seeking behaviour of the target audience and sociocultural and contextual barriers) and the supply side (e.g., setting, providers and supplies) [5]. For example, to ensure the efficient use of HIV-testing, operational issues can be addressed by evaluating various HIV-testing approaches, such as routine testing versus voluntary testing, and the outcomes can vary according to purpose and target population [6,7].

Given the need to enhance effectiveness of a broad range of tools in a variety of contexts, OR uses a wide range of methodologies, often making it difficult to have uniform reporting in publications. As a way of addressing this challenge, the *Bulletin of the World Health Organization* established guidelines for reporting on OR, which include intervention frequency, duration and intensity [8]. Similarly, the UK Medical Research Council recently published guidance on conducting and reporting on studies testing public health interventions, recommending that studies provide data on intervention implementation, including provider training, fidelity, dose and adaptation [9].

However, the collection and reporting of process data may sometimes be seen as being in conflict with or a duplication of efforts to measure outcomes [9]. One approach that can ensure that the focus owed to intervention implementation is not lost is to view the study using the lens of programme evaluation, which includes formative assessment (to determine concept and design), followed by process evaluation (to monitor inputs and outputs) and effectiveness evaluation (to assess outcomes and effectiveness) [10]. Indeed, if researchers anticipate analyzing outcome data stratified by intervention exposure, they should pay more attention to how intervention process data is collected and reported [11].

The objective of this commentary is to review experiences in HIV OR intervention design, implementation, process data collection and publication in order to identify gaps, contribute to the body of knowledge and propose a way forward for improving the focus on “implementation” in implementation research. The purpose is to emphasize the need to devote as much time and resources to the intervention as are given to outcome measurement.

## Discussion

### Formative research

Given the diversity of the context in which real-life public health interventions are applied, it is important not to transfer interventions from one setting to another without adaptation based on formative research [12]. Formative research is usually carried out using qualitative methodologies among potential service recipients, potential providers and other stakeholders, as well as a review of retrospective statistics and epidemiological and behavioural data.

Formative research enables understanding of the nature of the problem and the programme responses to address it [1,13,14] and determination of the priority target population [15,16], as well as assessing their needs and perceptions regarding the problem and the proposed intervention. Formative research also fosters an understanding of how the new intervention will be introduced into existing services [6,17,18], the potential role of various actors in delivering the intervention and additional elements required for the intervention [19].

### Piloting the intervention

Pilot testing is a key component of intervention design in HIV OR because it enables the determination of the feasibility of the intervention and its acceptability to providers and clients, thus facilitating adaptation.

To start, a cross-section of providers and clients can be asked to comment on the intervention materials. Next, the service should be offered on a small scale to obtain provider and client feedback [14]. There is also a need to be flexible and open-minded to accommodate contextual issues, including resource constraints while, at the same time, ensuring that the core elements of the intervention are retained [17]. Although the retention of core elements is vital to ensure that the intervention can be replicated and scaled up in other settings [20], there is always a tension between the need for standardization of the intervention versus the need to be flexible and adapt the intervention based on findings from pilot testing [17].

### Integration and training

When conducting OR, systematic efforts are required to ensure that new interventions are tailored to the realities of local settings [21] and ensure acceptance by existing providers [22]. This process could involve meetings with government authorities, managers, providers and other stakeholders to create awareness and ownership of the planned OR, work out operational issues and clarify roles among the providers [3,23–25].

In addition, the integration of a new intervention must involve training the existing providers in the delivery of the intervention according to procedures and curricula developed or adapted using formative findings [21,26]. After the study is completed, intervention training manuals, as well as videos and other written materials, should be finalized and made accessible to policy makers, programme developers and researchers [27].

In a systematic review of integration of sexual and reproductive health and HIV services, it was reported that integration showed positive effects on HIV incidence, sexually transmitted infections incidence, condom use, the uptake of HIV testing and quality of services. Facilitating factors included stakeholder involvement, capacity building and positive staff attitudes. Inhibiting factors included lack of stakeholder commitment and inadequate staff training, thus highlighting gaps in stakeholder engagement and staff training [28].

### Intervention dose

Using inputs from formative research, pilot testing and the process of integration, the “dose” of the intervention, such as the number of counselling sessions, may be developed or refined. In addition, standard operating procedures (SOPs) of service delivery are developed to ensure that the intervention is delivered as planned [14,19,29]. Low-intervention exposure has been proposed as one of the reasons for “flat” results in intervention trials despite a strong theoretical basis for the potential impact of an intervention [3,29,30]. Thus, it becomes important that studies accurately report intervention exposure. Owing to inconsistency in the reporting of intervention doses, a group of authors have proposed a standardized terminology for intervention dosing, including duration, frequency and amount, that should be used for reporting [27].

In a meta-analytic review of 19 studies of highly active antiretroviral therapy (HAART) adherence interventions, the number of intervention sessions and their durations varied widely. However, even though overall the interventions were effective in increasing adherence and reducing viral load, there was no difference in outcome by dose [31]. A review of 24 antiretroviral therapy (ART) adherence studies of the interventions also showed an overall positive effect on self-reported adherence, but no variation by duration of exposure. These reviewers were disappointed that the authors did not publish sufficient information about intervention characteristics to enable further exploration of factors beyond duration of exposure [32].

A similar finding was observable in a meta-analysis evaluating the efficacy of HIV behavioural interventions among African-American women, in which it was noted that the efficacy did not differ by the number of sessions. The authors suggested that the success of such interventions may depend more on the intervention components and quality than on the number of sessions [33]. However, in one study among HIV-positive women, it was observed that women who attended eight or more sessions of the intervention reported higher ART adherence [34]. Thus, gaps in reporting of intervention characteristics make it difficult to meaningfully assess the role of the intervention dose and other factors in the outcomes observed. Such an assessment requires detailed process data because the intervention dose may be affected by a multiplicity of aspects. This includes internal factors, such as fidelity, as well as external factors in the target community and beyond, among them an enabling environment and such issues as the weather, politics or population migrations [35].

### Uptake and coverage

Though the ultimate purpose of OR may be to determine the outcome of the intervention, it is important that process data is collected to determine the proportion of the targeted population that used the intervention. If the study is measuring outcomes of the intervention among clients receiving a clinic service, the proportion of clients who partook of the intervention among those who attended the clinic could serve as a good measure of intervention uptake and reach [19]. However, if the study is interested in community-level impact, coverage would be a more appropriate measure, and it could be assessed against a denominator of the estimated size of the target population, such as the number of drug users in a city [36] or the number of HIV-positive pregnant women in a district [6].

### Fidelity

Fidelity is the extent to which the provider delivers the intervention according to the set SOPs. Fidelity can be measured using client interaction forms[31,32], observation of selected client provider interaction sessions [14], interviews of providers and clients and the use of mystery clients.

In a behavioural programme targeting young people, fidelity was measured using a telephone survey of providers. The results showed varying levels of fidelity. Time constraints were commonly cited as reasons for dropping core elements [37]. In one study of a clinician-delivered HIV risk-reduction intervention, fidelity was measured through clinician self-reports and client exit interviews; it was observed that there was a convergence of opinion between clinicians and patients that only 73% of the intervention was delivered. Other pressing medical priorities are the main explanation for the failure to deliver 100% of the intervention. In spite of the less-than-perfect fidelity, the intervention was effective in reducing high-risk sexual behaviour [22].

In a systematic review of HIV-prevention interventions for young people, most of which were delivered by either teachers, peers or media, no positive effects of the intervention were observed. This was attributed to implementation barriers, including disorganized school schedules and the reluctance of teachers to discuss condoms. The authors recommended devoting more effort to studying implementation difficulties and the determinants of exposure to intervention [11].

Indeed, positive attitudes of providers are important to ensure fidelity. In an evaluation of the factors affecting fidelity in a school HIV education intervention, it was observed that teachers’ comfort with the HIV curriculum was the most important predictor of fidelity to the programme [38]. When assessing the rollout of evidence-based HIV prevention interventions, it was observed that the main factors hindering fidelity were a lack of adequate funding, staff and other resources [35,39]. In one study evaluating antenatal care counselling, 203 counselling sessions were observed, and it was reported that counselling sessions were shorter and conveyed fewer messages than required in the SOPs of the intervention [24], emphasizing the need to assess what was delivered versus what was supposed to be delivered [27].

## Conclusions

Interventions in OR, like ordinary service delivery programmes, are subject to the programme cycle, which continually uses insights from implementation and from the local context to modify service delivery modalities. Given that some of these modifications in the intervention may influence study outcomes, the documentation of process data becomes vital for understanding factors influencing the outcomes. However, a key issue is that study resources appear to be skewed towards the measurement of baseline and end-line, often relying on the use of routine service delivery statistics for process data [17]. It is also a reality that service delivery in low-resourced settings is plagued by the endemic lack of adequate funding, staff and other resources [5,39,35], resulting in serious gaps in routine data making the data difficult to use in research [40]. OR study protocols should include plans and budgets for process data collection, in addition to baseline and end-line surveys, and not rely only on service delivery statistics [16].

Since the purpose of OR is to test and roll out interventions that enhance the efficient use of proven tools for HIV prevention (e.g., condoms), treatment (e.g., ARVs) and diagnosis (e.g., HIV tests), it is incumbent upon the research team to document and publish the processes and materials used in implementation. This will help answer the questions: “If it worked, what worked?” and “How can it be replicated?” Intervention materials are an important legacy of a good OR study. Mechanisms for accessing these materials, preferably via the Internet, should be clearly stated in the outcome publication of such a study.
